# A Combination of Histological, Physiological, and Proteomic Approaches Shed Light on Seed Desiccation Tolerance of the Basal Angiosperm *Amborella trichopoda*

**DOI:** 10.3390/proteomes5030019

**Published:** 2017-07-28

**Authors:** Matthieu Villegente, Philippe Marmey, Claudette Job, Marc Galland, Gwendal Cueff, Béatrice Godin, Loïc Rajjou, Thierry Balliau, Michel Zivy, Bruno Fogliani, Valérie Sarramegna-Burtet, Dominique Job

**Affiliations:** 1 Institut des Sciences Exactes et Appliquées (EA 7484), Université de Nouvelle-Calédonie, BP R4, 98851 Nouméa, Nouvelle-Calédonie; mvillegente@gmail.com (M.V.); valerie.sarramegna@univ-nc.nc (V.S.-B.); 2 Institut de recherche pour le développement (IRD), UMR Diversité, Adaptation et Développement des plantes (DIADE), BP A5, 98848 Nouméa Cedex, Nouvelle-Calédonie; 3 Centre National de la Recherche Scientifique (CNRS), CNRS-Université Claude Bernard Lyon-Institut National des Sciences Appliquées-Bayer CropScience (UMR5240), Bayer CropScience, F-69263 Lyon CEDEX 9, France; claudette.job@gmail.com; 4 IJPB, Institut Jean-Pierre Bourgin (Institut National de la Rechercherche Agronomique(INRA), AgroParisTech, CNRS, Université Paris-Saclay) ; « Saclay Plant Sciences (SPS) » - RD10, F-78026 Versailles, France; mgalland1983@gmail.com (M.G.); gwendal.cueff@agroparistech.fr (G.C.); beatrice.godin@versailles.inra.fr (B.G.); loic.rajjou@agroparistech.fr (L.R.); 5 AgroParisTech, Département « Science de la Vie et Santé », Unité de Formation-Recherche en Physiologie végétale, F-75231 Paris, France; 6 Plateforme d'Analyse Protéomique de Paris Sud Ouest (PAPPSO), GQE–Le Moulon, INRA, Université Paris-Sud, CNRS, AgroParisTech, Université Paris-Saclay, F-91190 Gif-sur-Yvette, France; balliau@moulon.inra.fr (T.B.); zivy@moulon.inra.fr (M.Z.); 7 Institut Agronomique Néo-Calédonien (IAC), Équipe ARBOREAL, Agriculture Biodiversité et Valorisation, BP 73 Port Laguerre, 98890 Païta, Nouvelle-Calédonie

**Keywords:** *Amborella trichopoda*, basal angiosperms, seeds, desiccation tolerance, proteomics

## Abstract

Desiccation tolerance allows plant seeds to remain viable in a dry state for years and even centuries. To reveal potential evolutionary processes of this trait, we have conducted a shotgun proteomic analysis of isolated embryo and endosperm from mature seeds *of Amborella trichopoda*, an understory shrub endemic to New Caledonia that is considered to be the basal extant angiosperm. The present analysis led to the characterization of 415 and 69 proteins from the isolated embryo and endosperm tissues, respectively. The role of these proteins is discussed in terms of protein evolution and physiological properties of the rudimentary, underdeveloped, *Amborella* embryos, notably considering that the acquisition of desiccation tolerance corresponds to the final developmental stage of mature seeds possessing large embryos.

## 1. Introduction

*Amborella trichopoda*, the eldest sister of all flowering plants, lies at the base of the phylogenetic tree of angiosperms [1,2,3,4,5], and it would have emerged about 135 Ma ago. This plant is thus of great interest to the scientific community, as evidenced by the recent completion of its genome sequence [6]. It is a dioecious shrub, 2–6 m high, which is endemic to New Caledonia and grows at medium altitude (400–800 m) in wet forests. Several studies have been carried out on this species to reveal plesiomorphic (primitive) characters, notably concerning its vegetative anatomy [7], its fruits [8,9,10,11], the very special structure of its vascular system that does not possess xylemic vessels [12], or the evolution of gene families [6,13].

The study by Tobe et al. [9] disclosed for the first time the existence of a very small heart-shaped embryo in *Amborella* mature seeds. These observations are in agreement with the idea that an underdeveloped embryo is the most primitive form in seeds, although small embryos are also found in non-basal seed species (e.g., celery, tomato) [14,15,16,17,18,19]. Indeed, the ratio (E:S) between the length of the embryo (E) and that of the seed (S) increases during evolution [17]. Seeds with a rudimentary embryo have morphological dormancy, which is considered the most primitive dormancy [20,21,22]. Consequently, such seeds can only germinate after continuation of the embryo development during seed imbibition after dispersal from the mother plant. This type of dormancy is found in all the families of the ANITA basal grade (composed of *Amborella*, *Nymphaeales* and *Illiciales* and *Trimeniaceae*-*Austrobaileya*) except for the *Nymphaeales*, and also in some more modern species of mono- and eudicotyledons.

In orthodox seeds, desiccation tolerance allowing seeds to survive in the dry state is acquired very late in development. Given the underdeveloped nature of the *Amborella* embryo, this may suggest temporal differences in expression of the genes and proteins involved in desiccation tolerance between basal and modern orthodox seed species. In the present work, we used a combination of physiological and proteomics approaches to address various questions concerning the state of maturity of the *Amborella* embryo present in mature seeds, in particular with regard to desiccation tolerance.

## 2. Materials and Methods

### 2.1. Fruits, Seeds, and Embryos

Mature *Amborella* drupes were collected at a mature stage [23] from individual trees located at “Plateau de Dogny-Sarraméa” in the central mountain range of New Caledonia. The fleshy part of the fruits was removed before *sensu stricto* seed isolation. For embryo isolation, surface-sterilized seeds were longitudinally cut in two with a razor blade [22]. A drop of sterile Milli-Q water was placed on the endospermic face on each half. Embryos were quickly extracted (in <1 min), with extra-thin needles and immediately frozen in liquid nitrogen [13].

### 2.2. Histochemistry

#### 2.2.1. Inclusion in Historesin

*Sensu stricto* seeds were placed in a fixative medium (paraformaldehyde 2% (*w/v*)/glutaraldehyde 1% (*v/v*)/caffeine 1% (*w/v*), 0.05% Triton X-100 in sodium phosphate buffer pH 7.2; four successive infiltrations with the aid of a vacuum pump) and incubated overnight at 4 °C with slow circular stirring. After fixation, seeds were dehydrated gradually with slow circular stirring using a series of solutions corresponding to 50% ethanol (EtOH) for 30 min, 70% EtOH for 30 min, 70% EtOH for 1 h, 95% EtOH twice for 30 min, 100% EtOH for 1 h, EtOH/butan-1-ol (1:1, *v/v*) for 1 h, 100% butan-1-ol overnight at 4 °C. After dehydration, seeds were impregnated (2*v*/2*v*) for 24 h at 4 °C, butan-1-ol/Historesin Technovit (2*v*/2*v*) for 24 h at 4 °C, butan-1-ol/An-1-ol/Historesin Technovit (1*v*:3*v*) for 24 h at 4 °C, Historesin Technovit 100%. *Sensu stricto* seeds were then included in 1 mL of Historesin inclusion solution (Historesin/Hardening 15:1) at room temperature for 1 h and then at 37 °C for at least two days. Seeds were then cut using a microtome set at 5 μm thickness.

#### 2.2.2. Inclusion in Agarose

*Sensu stricto* seeds were fixed in low-melting agarose (Sigma-A9414, St Louis, MO, USA) when the temperature of the solution was below 40 °C. Solidification was obtained at room temperature for 4 h.

#### 2.2.3. Double Staining with Naphthol Blue Black and Periodic Acid-Schiff Reagent

This double staining allows the simultaneous visualization of polysaccharide networks (Schiff’s periodic acid reagent) [24] and protein and nucleolous bodies (Naphthol blue black (Sigma, St Louis, MO, USA)) [25]. Sections obtained from seeds fixed in the resin were hydrolyzed 5 min in periodic acid (1%) and then rinsed with distilled water. The first Schiff staining was carried out for 10 min in the dark. Sections were then rinsed twice in sulfuric acid (0.25% sodium metabisulfite, 0.05 M hydrochloric acid) and then with running water and distilled water until the washing water was clear. Sections were then treated for 5 min with the solution of Naphthol blue black previously heated at 60 °C (1% Naphthol black blue in 7% acetic acid) and then rapidly rinsed with running water. Regressive staining was carried out with acetic acid (7%) under a microscope (Zeiss Axioplan optical microscope, Zeiss, Mannheim, Germany).

#### 2.2.4. Staining with Nile Red 

Nile red allows the specific staining of lipid bodies [26]. *Sensu stricto* seeds included in the agarose were cut longitudinally using a vibrating blade microtome (Leica VT1000S, Leica Microsystemes SAS, Nanterre, France), adjusted to a thickness of 30 μm. The working solution of Nile red was prepared extemporaneously by diluting the stock solution a hundred-fold (1 mg/mL Nile red in 100% acetone) in 50% (*v/v*) glycerol. Sections were incubated for 30 min in the dark and then directly placed between the blade and the slide in a drop of water. Observations were carried out using a Leica TCS SP2 laser scanning confocal microscope (Leica Microsystemes SAS, Nanterre, France) with excitation at 485 nm and emission at 525 nm.

### 2.3. Seed Desiccation Tolerance

Fleshy parts of drupes were removed to obtain seeds. Seeds of a given batch were from the same shrub; they were all scarified by soaking them in a solution of sulfuric acid for 20 min and then rinsed several times with water [13,27]. Seed batches were desiccated by equilibration for one week at 25 °C using different saturated salt solutions [28] The nine saturated salt solutions, namely KOH, C_2_H_3_KO_2_, MgCl_2_, K_2_CO_3_, NH_4_NO_3_, NaCl, (NH_4_)_2_SO_4_, KCl, and KNO_3_, allowed to obtain a range of nine different relative humidities (RHs), with values of 9%, 23%, 34%, 45%, 62%, 75%, 81%, 85%, 92%, respectively. A sorption isotherm was established using all saturated salt solutions, with a total of ten seeds per RH. A desiccation experiment consisted of ten batches of 50 seeds (all from the same shrub), nine of which corresponding to the nine above-mentioned RHs, and one batch, as a control that was scarified by sulfuric acid too but with no treatment for desiccation. All seed batches were then sown in a mixture of potting soil and perlite (50/50) and placed in a greenhouse with controlled humidity and on a heated (19 °C) bench in winter time. Statistical analysis using GraphPad-PRISM Software (version 5.00, La Jolla, San Diego, CA, USA) was performed to analyze germination data.

### 2.4. Preparation of Protein Extracts

Protein extraction was carried out from three replicates of 100 isolated embryos and three replicates of 20 endosperm portions (free of embryos). Proteins were extracted at room temperature in 400 μL thiourea/urea lysis buffer composed of 7 M urea, 2 M thiourea, 6 mM Tris-HCl, 4.2 mM Trizma^®^ base (Sigma, St Louis, MO, USA), 4% (*w/v*) 3-[(3-cholamidopropyl)dimethylammonio]-1-propanesulfonate (CHAPS, Sigma, St Louis, MO, USA) supplemented with 50 µL of the protease inhibitor cocktail Complete Mini (Roche Diagnostics, Mannheim, Germany)). Then, 15 µL of 1 M dithiothreitol (DTT, Sigma-Aldrich, St Louis, MO, USA), 2 µL of DNase I (Sigma, St Louis, MO, USA) and 5 µL of RNase A (Sigma, St Louis, MO, USA) were added to the sample. Following stirring for 2 h at 4 °C, protein extracts were centrifuged at 20,000 *g* at 4 °C for 15 min. The resulting supernatant was submitted to a second clarifying centrifugation, as above [13,29]. The final supernatant was kept and protein concentrations in the various extracts were measured using bovine serum albumin as a standard [30].

### 2.5. Shotgun Proteomics 

The *Amborella* seed proteome exploration was performed as previously reported [13] by LC-MS/MS analysis following preparation of soluble protein extracts (30 µg protein; *n* = 3 biological replicates) that had been subjected to 1D-SDS-PAGE (http://pappso.inra.fr). Protein extracts were loaded in 1X Laemmli buffer with DTT (50 mM) in a stacking gel (acrylamide 8%; Tris-HCl 563 mM, pH 8.8, SDS 0.1% (*w/v*)). After 15 min of migration at 10 mA, the gel was stained with colloidal blue (GelCode Blue Stain Reagent; Thermo Fisher Scientific Inc, Rockford, IL, USA) and destained in Milli-Q water. The whole band corresponding to total proteins was excised and submitted to in-gel digestion with the Progest system (Genomic Solution, Huntingdon, UK) according to a standard trypsin protocol. Briefly, gel pieces were washed for 1 h at 37 °C in a solution containing 25% (*v/v*) acetonitrile and 50 mM ammonium bicarbonate (pH 7.8), followed by dehydration in 100% acetonitrile (ACN) for 15 min. Gel pieces were rehydrated overnight at 37 °C with 1/50 (*w/w*) trypsin (Promega, Madison, WI, USA) in 20 mM ammonium bicarbonate, pH 7.8. Digestion was stopped by adding 0.4% of trifluoroacetic acid (TFA). 

HPLC was performed on a NanoLC-Ultra system (Eksigent, Les Ulis, France)). A 4-µL sample was loaded at 7.5 µL/min^−1^ on a precolumn cartridge (stationary phase: BIOSPHERE C18, 5 µm; column: 100 µm i.d., 2 cm; NanoSeparations, Nieuwkoop, The Netherlands) and desalted with 0.1% methanoic acid (HCOOH). After 3 min, the precolumn cartridge was connected to the separating PepMap C18 column (stationary phase: BIOSPHERE C18, 3 µm; column: 75 µm i.d., 150 mm; NanoSeparations). Buffers used were 0.1% HCOOH in water (A) and 0.1% HCOOH in ACN (B). Peptide separation was achieved with a linear gradient from 5 to 30% B for 30 min at 300 nL/min. Including the regeneration step at 95% B and the equilibration step at 95% A, one run took 45 min. Eluted peptides were analyzed on-line with a Q-Exactive mass spectrometer (Thermo Electron, Waltham, MA, USA) using a nano-electrospray interface (non-coated capillary probe, 10 µ i.d.; New Objective, Woburn, MA, USA). Xcalibur 2.1 interface was used to monitor data-dependent acquisition of peptide ions. This acquisition included a full MS scan covering 300 to 1400 range of mass-to-charge ratio (*m/z*) with a resolution of 70,000 and a MS/MS step (normalized collision energy: 30%; resolution: 17,500). MS/MS step was reiterated for the eight major ions detected during full MS scan. Dynamic exclusion was set to 45 s. 

A database search was performed with X!Tandem [31] for protein identification. Enzymatic cleavage was declared as a trypsin digestion with one possible miscleavage. Cys carbamidomethylation and Met oxidation were declared as fixed and variable modifications, respectively. Precursor mass and fragment mass tolerance were 10 ppm and 0.02 Th, respectively. The *Amborella* Genome database (http://www.amborella.org/; “evm_27.model.AmTr_v1.0_scaffold00004.99”) and a contaminant database (trypsin, keratins) were used. Identified proteins were analyzed using X!TandemPipeline [32]. Only peptides with an E-value smaller than 0.05 were validated, and at least two valid peptides were required to validate a protein. Peptide sequences predicted from the *Amborella* genome were then submitted to BLAST analyses against the non-redundant protein sequences at NCBI, making it possible to evaluate their role in the physiology of the seed (http://metacyc. org). These analyzes allowed us to assign a function to the majority of the identified proteins. The functional categories and sub-categories are those defined in [33].

## 3. Results

### 3.1. Histochemistry

**Figure 1:**
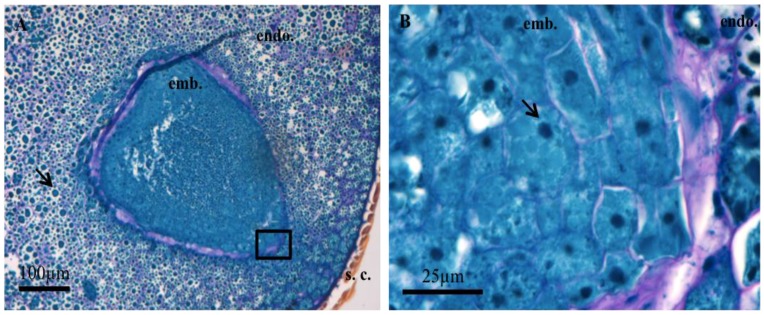
Optical microscope observations of sections of *Amborella* dry mature *sensu stricto* seeds after Naphthol blue black staining combined with periodic acid staining/Schiff. (**A**) embryo (emb.), Endosperm (endo.), Envelope (s.c.); (**B**) enlarged view of the area represented by the black rectangle in (**A**). The proteins and protein bodies are stained in blue (arrow A), the nucleoli in dark blue (arrow B) and the polysaccharides in pink.

**Figure 2:**
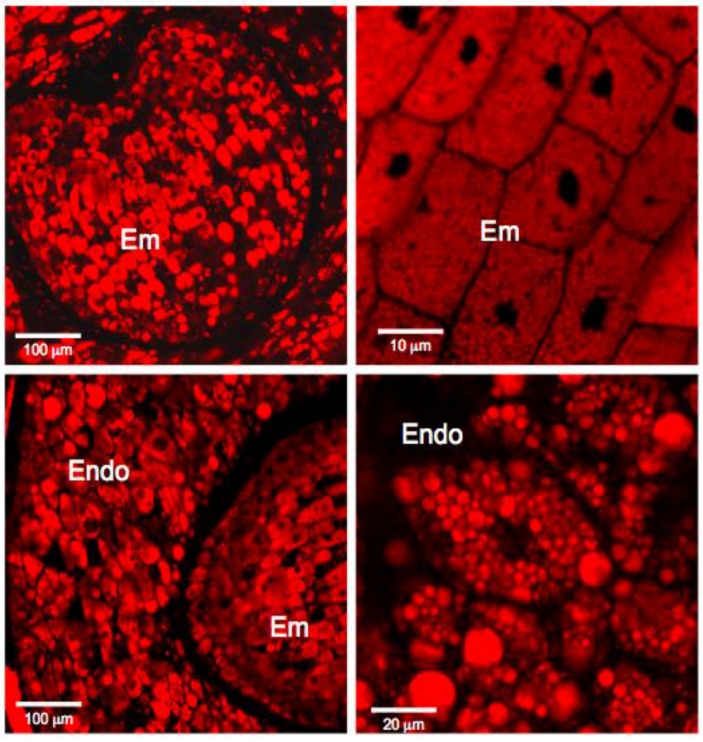
Confocal microscopic observation of *Amborella sensu stricto* seed sections following staining with Nile red showing lipid distribution in the embryo (Em) and the endosperm (Endo).

The dry mature *Amborella sensu stricto* seeds are composed of a bulky albumen, an envelope, and a rudimentary embryo of small size, eccentric, and heart-shaped (ratio embryo/seed length = 0.08). Seed cuts were stained with Naphthol blue black to visualize proteins (blue) and nuclei (dark blue). This staining also contained periodic acid/Schiff reagent to reveal the presence of polysaccharides (pink) (Figure 1). Embryonic cells contain a nucleus and protein bodies and are delimited by a very thin wall (pink coloration). The whole embryo is surrounded by a thick network of polysaccharides (stained in pink) in which dead cells are imbricated (Figure 1). Endosperm cells have walls that are more visible than those of the embryo and are larger (about 70 μm long, maximum 50 μm for the embryo) (Figure 1). They contain a nucleus and many protein bodies (Figure 1). An examination by confocal microscopy of seed sections stained with Nile red revealed the presence of lipid bodies [34,35], both in the embryo and in the endosperm (Figure 2). These observations raise questions about the maturity of the embryo and the endosperm. These issues have been addressed by physiological and proteomic approaches.

### 3.2. Desiccation Tolerance of the Mature Amborella Seeds

After harvest, *Amborella* seeds displayed a water content of 12.9% (in terms of fresh weight). These seeds (after scarification) displayed a germination percentage above 90%. It was interesting to determine the level of desiccation that seeds of this rainforest shrub could tolerate in order to confirm this trait for this basal angiosperm. For this purpose, desiccation tolerance experiments were carried out on *Amborella* seed batches.

**Figure 3:**
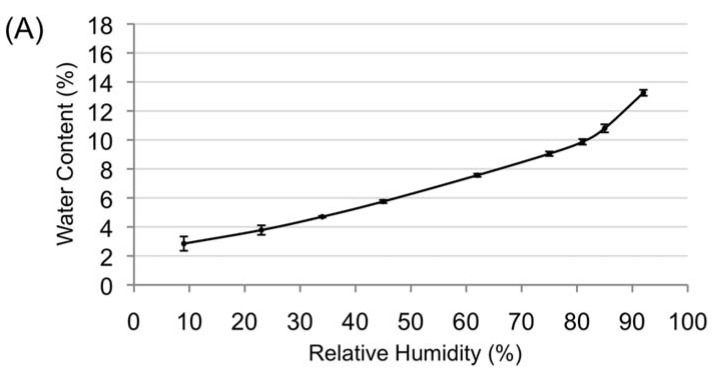

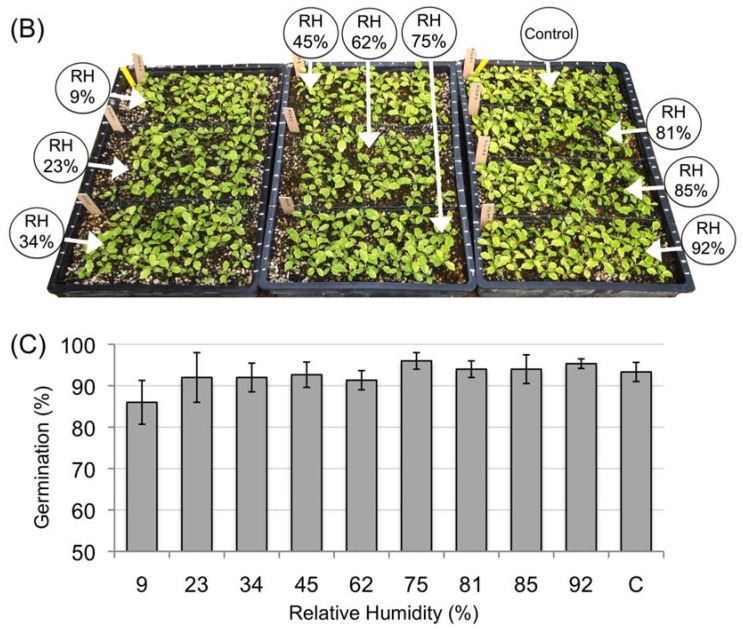
Desiccation tolerance of *Amborella* seeds. Desiccation was monitored using a range of atmospheres where relative humidities (RHs) are controlled using different saturated salt solutions. (**A**) Water sorption isotherm curve issued from three replicates made on *Amborella* seeds at 25 °C. The water content at different RH values is expressed on a fresh weight basis; (**B**) Observation of germination and seedling appearance at 119 days post sowing for seed batches issued from a desiccation experiment after equilibrium in nine different relative humidities (RH%) and a control (not desiccated); (**C**) Germination percentages measured for seed batches at 140 days post sowing issued from desiccation experiments after equilibrium in nine different RH values and a control seed sample (not desiccated). Percentages are issued from three replicates. Means ± standard deviations were not significantly different, as estimated by One-Way ANOVA followed by Bonferroni’s multiple comparisons test (with alpha value of 0.05).

To test the endocarp permeability toward moisture, non-scarified seed batches were placed in three different relative humidities (RHs), namely RH 9%, RH 45%, and RH 85%, and then weighed until equilibrium was reached (data not shown). Change of mass variation with respect to control seeds for each seed batch was −8.9%, −4.9%, and +0.5%, respectively. Endocarps were then removed from seeds, and *sensu stricto* seed batches were weighed and placed in same RHs as above. No change (less than 0.1%) of *sensu stricto* seed mass was noted in the next seven days for batches placed in the three studied RHs, reflecting the permeability of the endocarp and the action of desiccation on *sensu stricto* seeds. Equilibrium of seeds in the various studied RHs was reached after four days. Water sorption isotherms were drawn using moisture content of seeds equilibrated at all the studied RHs. The water sorption isotherm, issued from three replicates, showed typical curves with two regions observed above RH 9%, one ranging from 9–80%, the second above RH 80% (Figure 3A). The water content was 2.85 ± 0.5% for RH 9%, 9.87 ± 0.2% for RH 81%, and 13.25 ± 0.2% for RH 92% (Figure 3A).

Seed batches issued from three replicates of a desiccation experiment were put in germination in a greenhouse. Germination of seeds occurred 70 days after sowing, and the final rate of germination was obtained 140 days after sowing (Figure 3B). Germination percentage presented for each RH condition was the average of three replicates of a desiccation experiments, with a total of 50 seeds sown per RH and per experiment (Figure 3C). Mean of germination percentages of controls (non-desiccated seeds) was 93.3 ± 2.3%. Means of germination percentages of desiccated seed batches were 86.0 ± 5.3%, 92.0 ± 6.0%, 92.0 ± 3.5%, 92.7 ± 3.1%, 91.3 ± 2.3%, 96.0 ± 2.0%, 94.0 ± 2.0%, 95.3 ± 1.1%, and 93.3 ± 2.3% for RH of 9, 23, 34, 45, 62, 75, 81, 85, and 92%, respectively. When results were expressed in germination percentage of corresponding controls, the mean of germination percentages was 92.1 ± 3.4% for seeds batches equilibrated in RH 9%. Statistical analysis performed using one-way ANOVA and Bonferroni’s multiple comparisons test revealed that means of germination of seeds for the nine conditions of desiccation plus controls were not significantly different (with *p* < 0.05).

### 3.3. Characterization of the Amborella Seed Proteins by Shotgun Proteomics 

**Figure 4:**
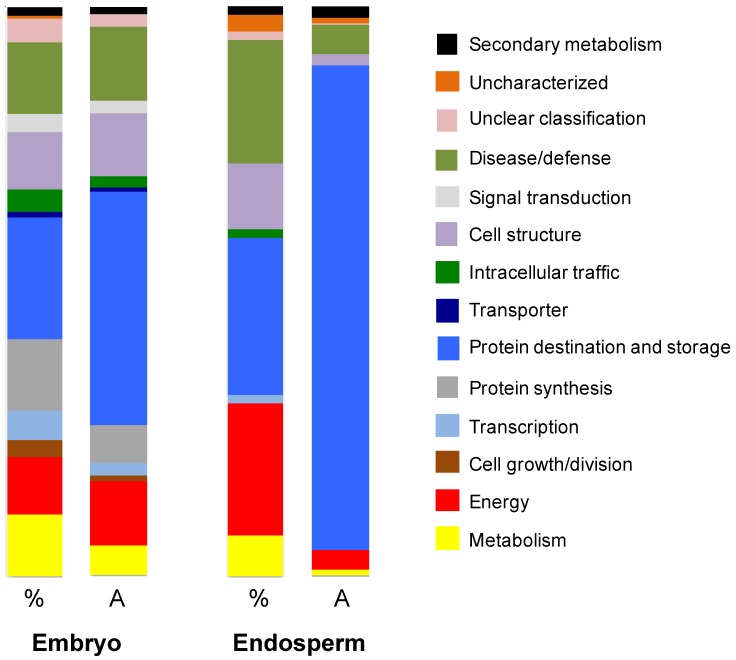
Representation of the relative importance of the ontological classes [33] of the proteins extracted from the embryo and endosperm of *Amborella sensu stricto* seeds. The results are expressed as a function of the number of proteins identified by class in relation to the total number of proteins identified (%) or according to their relative quantities among the identified proteins (A).

This analysis was carried out using (i) 300 embryos isolated from dry mature *sensu stricto* seeds and (ii) from 20 portions of endosperms without embryos (1.2 g) also isolated from dry mature *sensu stricto Amborella* seeds (see “Materials and Methods,” [13]). A shotgun proteomic approach was favored because this sensitive technique is particularly suitable for samples available in very small quantities [36], as is the case for *Amborella* embryos. The extraction of total proteins allowed identifying 69 proteins from the isolated endosperm (Supplementary Table S1) and 415 proteins from the isolated embryo (Supplementary Table S2). The identified proteins were then grouped according to their ontological class and description [33]. The representation of each category and function was expressed as a percentage of the total number of proteins identified, as well as the relative amount of each identification (abundance; see Materials and Methods). This estimate was made from the number of peptides corresponding to each identification (Figure 4; Supplementary Tables S1 and Tables S2). Among the 69 proteins identified from the endosperm, 12 were not detected in the embryo (proteins labelled Endo in Supplementary Table S1). 

#### 3.3.1. Endosperm Proteins 

The 69 identified proteins identified from the endosperm correspond to 20 unique functions. They are grouped into major categories (Figure 4) in relation to their relative abundance (Supplementary Table S1). The *Protein destination and storage* category contains 19 proteins representing 85.1% of the abundance of all proteins. Eight proteins are of the *Storage protein* function solely representing 82.6% in total protein abundance. The *Folding and stability* function contains seven proteins (1.8% in abundance). Fifteen proteins are in the *Disease/Defense* category (5.1% in total abundance). The five proteins of the *Stress response* function represent 2.4% in abundance. The 11 proteins of the *Detoxification* function represent 4.6% in abundance. The 16 proteins in the *Energy* category represent 3.4% in abundance. These are mainly involved in three main functions: glycolysis, tricarboxylic acid (TCA) pathway cycle and fermentation. The five proteins involved in the *Metabolism* category represent 1% of abundance. The *Cell structure* category (eight proteins) represents 1.9% of abundance. These results clearly demonstrate that the endosperm is primarily a tissue for storage of seminal reserves, as highlighted by a relative abundance of storage proteins of about 82%.

#### 3.3.2. Embryo Proteins

The ontological classification of the 415 proteins identified in the embryo is radically different (Figure 4; Supplementary Table S2). They correspond to 52 unique functions. Eighty-nine proteins in the *Protein destination and storage* category account for 41.3% in abundance. Among them, eight are storage proteins that represent 25.5% of total abundance, which is about 3 times less than in endosperm. The *Metabolism* category contains 45 proteins (5.3% in abundance). Eighteen proteins are involved in the *Amino acid* function (1.6% of total abundance). Fifteen proteins are involved in the *Sugars and polysaccharides* function (2% of the proteins in abundance). Five proteins are involved in the *Lipid and sterol* function (0.9% in abundance). The functional category *Energy* contains 42 proteins and represents an abundance of 11.1%. In particular, the *Glycolysis* function contains 18 proteins (6.7% in abundance). The *TCA pathway* function is represented by 14 proteins (1.8% of the total protein abundance). There are 52 proteins in the *Protein synthesis* category, which represents 6.5% of the proteins of the embryo in terms of relative abundance.

In summary, besides a storage role the proteins of the embryo are associated with cellular mechanisms (Figure 4).

## 4. Discussion

In agreement with previous observations [9,22], the present results show that the small *Amborella* embryo is surrounded by a voluminous endosperm (Figure 1), which is characteristic of the seeds of basal plants [17,20,22]. We also show, for the first time, that the embryo is surrounded by a network of polysaccharides interspersed with a network of empty, dead cells (Figure 1). Such a network has not been previously observed in seeds of other basal *species*, including *Trimenia austinensis*, *Trithuria submersa*, *Trithuria cowieana*, *Trithuria lanterna*, *Nymphaea lotus*, or *Hydatella inconspicua* [37,38,39]. On the other hand, the presence of dead cells surrounding the embryo has been described for celery seeds that also contain a very small embryo within a bulky endosperm [14]. It is known that following imbibition, the *Amborella* embryo develops in the seed after dispersal from the mother plant before germination *sensu stricto*, as occurs for celery [14,22]. The presence of this network of polysaccharides and dead cells could thus represent a structural evolution allowing to protect the embryo from the mechanical pressure of the endosperm and allowing its own differentiation in the seed. Upon staining with Naphtol blue black, both the embryo and endosperm cells exhibit protein bodies (Figure 1).

The level of seed desiccation tolerance had never been investigated in *Amborella*. To address this question, great attention was paid to the physiological experiments, with an optimal time of nine days between the harvest of fruits and the sowing of the desiccated seeds. All these stages took place in New Caledonia. Fruits for desiccation experiments were collected when the color of the exocarp turned red. This stage of maturity was the one for which the germinative capacity had been shown to be the highest [23]. The permeability of the endocarp was checked to ensure the desiccation effect on the *sensu stricto* seed. The results corroborated previous observations on the permeability of the *Amborella* endocarp [22]. The initial water content of *Amborella* seeds was 12.9% in terms of fresh weight. Seeds with the lowest water content, namely 2.85% fresh weight and corresponding to an equilibrium at 9% RH, displayed a germination rate of 86.0 ± 5.3%, which is not significantly different from the rate of non-desiccated seed controls (93.3 ± 2.3%). Thus, the present results reveal that a loss of more than 75% of their water content (in terms of fresh weight) does not significantly affect the germination vigor of *Amborella* seeds, attesting to their competence to withstand intense desiccation stress.

In orthodox seeds, the acquisition of desiccation tolerance occurs during the reserve accumulation phase [40,41]. The present shotgun proteomic analysis revealed that 82.6% (in abundance) of the proteins of the endosperm are storage proteins. These same proteins do also accumulate significantly in the embryo (25.5% abundance) albeit to an about three-fold lower level than in the endosperm (compare Supplementary Tables S1 and Tables S2). These results are in perfect agreement with the cytological observations showing the presence of numerous protein bodies in both the endosperm and the embryo (Figure 1). The current study also disclosed that the *Amborella* mature dry seed possesses a number of proteins necessary for the stabilization of the lipid storage bodies during desiccation. In particular, five oleosins were identified in the embryo, and one was present in both the endosperm and the embryo (Supplementary Tables S1 and Tables S2). These proteins, which stabilize lipid bodies, are found both in angiosperms and gymnosperms [42]. Their main role is to prevent the coalescence of the lipid bodies at the time of desiccation during late seed maturation, but also during seed imbibition and germination [43]. It is interesting to note that oleosins are present in a much lower amount in desiccation-sensitive recalcitrant seeds compared to desiccation-tolerant orthodox seeds [43,44]. The identification of these proteins in the *Amborella* seeds is therefore a good indicator of their ability to tolerate the desiccation stress by the end of seed maturation. It is established that during germination, the triacylglycerols (TAGs) are degraded into fatty acids which are then used for β-oxidation and then within the glyoxylate cycle [45,46,47]. In this context, it is noted that most enzymes of the β-oxidation pathway (with the exception of acyl-CoA oxidase) and the glyoxylate cycle (with the exception of isocitrate lyase) are present in the *Amborella* embryo (Supplementary Table S2). Interestingly both the activities of acyl-CoA oxidase and isocitrate lyase have been shown to specifically increase during seed imbibition [48,49].

The present proteomics analysis shows that about 11% of the embryo proteins are involved in the *Metabolism* functional category. Moreover, a number of these proteins are involved in the *Amino acids metabolism*, and in particular in the Met metabolism (Supplementary Table S2). It is well established that the Met metabolism is crucial for all living organisms. In plants, this amino acid not only serves as a building block for protein synthesis but it also supports vital metabolic functions as the methylation of proteins, nucleic acids and a myriad of metabolites, the synthesis of ethylene, a phytohormone, and the synthesis of biotin, a vitamin cofactor of several cellular carboxylases [50,51,52]. In the context of this sulfur metabolism, it is of interest to note that an enzyme called protein-l-isoaspartate-*O*-methyltansferase (PIMT) is present in the *Amborella* embryo (Supplementary Table S2). To our knowledge, this is the first identification by proteomics of this enzyme in plants. This protein is of paramount importance to preserve the functional integrity of the cellular proteome [53,54,55], notably in the repair of proteins damaged during aging. PIMT is a methyltransferase capable of catalyzing the conversion of abnormal l-iso-Asp residues generated during aging to their non-deleterious l-Asp form, using *S*-adenosyl-methionine as co-substrate [56]. In plants, this enzyme is directly linked to the preservation of seed vigor that might be altered during dry storage [56]. Thus, the overexpression of the PIMT enzyme in the seeds of *A. thaliana* increases their vigor (viability), whereas the opposite is observed by underexpression [56]. It has been proposed that the exceptional longevity of sacred lotus (*Nelumbo nucifera*) seeds is at least partly due to the extraordinary accumulation of PIMT activity within seed tissues, representing the highest accumulation ever observed in the living kingdom [57,58,59]. The identification of the PIMT enzyme in the *Amborella* embryo therefore suggests that the embryo is capable of repairing its damaged proteome during dry storage [29,60].

Three enzymes of the proline metabolism are present within the embryo, namely the P5C (Δ^1^-pyrroline-5-carboxylate) reductase, P5C dehydrogenase, and acetylornithine deacetylase (Supplementary Table S2) [61,62]. Proline is an osmoprotectant involved in the response to a number of abiotic stresses in plants, notably osmotic stress in seeds [63]. Glycine betaine is another important osmolyte involved in osmotic stress response in plants [64,65]. It is interesting to note that betaine aldehyde dehydrogenase, which is the terminal enzyme of glycine betaine synthesis, is present in the embryo (Supplementary Table S2).

The embryo also exhibits proteins involved in secondary metabolism, notably in the metabolism of plant defense reactions. Thus, this study revealed an isochorismatase (Supplementary Table S2), an enzyme that hydrolyzes isochorismate, a precursor of salicylic acid [66]. The identification of this enzyme could therefore suggest a potential inhibition of this biosynthetic pathway in the *Amborella* embryo. Moreover, the identification of a tocopherol *O*-methyltransferase (Supplementary Table S2) reveals the ability of the seed to synthesize α-tocopherol, which is a powerful molecule trapping oxidizing species, this process being vital for seed longevity and vigor [67,68]. It is also noted that the Mother of FT and TFL1 protein is detected in the *Amborella* embryo (Supplementary Table S2). This protein plays a crucial role in germination by exerting negative feedback on signaling by abscisic acid (ABA), a phytohormone behaving as a germination inhibitor [69]. The function of this protein seems to be conserved in plants, including bryophytes, which are the ancestors of all terrestrial plants [70]. Finally, the present data show that farnesylcysteine lyase, which is involved in negative regulation of ABA signaling in plants [71], is present in the embryo of *Amborella* seeds (Supplementary Table S2). Altogether, the present results are therefore in perfect agreement with these findings and suggest that the mechanisms involved in the regulation of seed maturation/germination controlled by ABA are present in *Amborella*.

Other proteomic data obtained in the present study are also in agreement with the acquisition of desiccation tolerance by the *Amborella* seed. Thus, both the embryo and the endosperm were shown to contain an arsenal of chaperone proteins (Supplementary Tables S1 and Tables S2), the function of which is to assist other proteins in their maturation, ensuring proper three-dimensional folding, notably under desiccation stress in seeds. These include proteins called Late Embryogenesis Abundant (LEA) and Heat Shock Proteins (HSP) that protect macromolecular complexes from stresses such as desiccation, dry storage, and imbibition (Supplementary Table S2). LEA proteins specifically accumulate in seeds during late stages of maturation [72]. Their involvement in the response to stresses, in particular water stress, is very well established [73,74,75,76,77,78,79]. The small HSPs (sHSPs) form complexes with partially structured or unstructured proteins and prevent their complete denaturation [80], thus contributing to seed longevity [81]. In addition to these small HSPs, the *Amborella* embryo and endosperm do contain other higher molecular weight HSPs also involved in protein structuring, such as HSP70 and HSP101 that are known to accumulate under stress conditions [82,83,84]. The presence of these chaperone proteins in the *Amborella* seeds could thus contribute to their observed desiccation tolerance.

## 5. Conclusions

From a biochemical and molecular point of view, it appears that the *Amborella* embryo and endosperm possess all the tools necessary to tolerate desiccation stress occurring during the final phases of the maturation of orthodox seeds. These data are important because the origin of tolerance to desiccation during evolution is controversial. Indeed, this tolerance appears to be a complex character, requiring the interaction of many genetic factors [85]. A first study of 45 species concluded that recalcitrant seeds (non-tolerant to desiccation) were associated with ancestral-type ovaries. Indeed, orthodoxy was considered to correspond to the evolved character [86]. However, more recent studies of a larger number of species have come to the opposite conclusion [87,88], in particular for species of the Hydatellaceae family, which is considered to be one of the oldest flowering plant lines [39]. These observations are in agreement with the Dollo’s law of irreversibility, which states that evolution is not irreversible and that for very complex characters (such as desiccation tolerance), parallel origin is highly unlikely, whereas reversal is quite easy [89]. 

The present characterization of a number of proteins from the *Amborella* seeds made it possible, for the first time, to obtain the proteome of an undifferentiated embryo in a mature seed. These data indicate that, despite its rudimentary appearance, the *Amborella* embryo contains proteins usually associated with late stages of development (maturation phase) in orthodox seeds, including (i) the ability to accumulate stored reserves, both proteins and lipids and (ii) the ability to tolerate desiccation, a process that is of paramount importance in agriculture [90,91].

## Supplementary Materials

The following are available online at http://www.mdpi.com/2227-7382/5/3/19/s1
